# Quantitative susceptibility mapping in magnetically inhomogeneous tissues

**DOI:** 10.1002/mrm.30537

**Published:** 2025-05-01

**Authors:** Thomas Jochmann, Fahad Salman, Michael G. Dwyer, Niels Bergsland, Robert Zivadinov, Jens Haueisen, Ferdinand Schweser

**Affiliations:** ^1^ Institute of Biomedical Engineering and Informatics, Department of Computer Science and Automation Technische Universität Ilmenau Ilmenau Germany; ^2^ Buffalo Neuroimaging Analysis Center, Department of Neurology, Jacobs School of Medicine and Biomedical Sciences at the University at Buffalo The State University of New York Buffalo New York USA; ^3^ Department of Biomedical Engineering University at Buffalo, The State University of New York Buffalo New York USA; ^4^ Center for Biomedical Imaging, Clinical and Translational Science Institute University at Buffalo, The State University of New York Buffalo New York USA; ^5^ Department of Neurology Jena University Hospital Jena Germany

**Keywords:** chemical exchange, macroscopically nondipolar Larmor frequency shifts, microstructure, phase contrast, quantitative susceptibility mapping

## Abstract

**Purpose:**

Conventional quantitative susceptibility mapping (QSM) methods rely on simplified physical models that assume isotropic and homogeneous tissue properties, leading to artifacts and inaccuracies in biological tissues. This study aims to develop and evaluate DEEPOLE, a deep learning–based method that incorporates macroscopically nondipolar Larmor frequency shifts into QSM to enhance the quality and accuracy of susceptibility maps.

**Methods:**

DEEPOLE integrates the QUASAR model into a deep convolutional neural network to account for frequency contributions neglected by conventional QSM. We trained DEEPOLE using synthesized data reflecting realistic power spectrum distributions. Its performance was evaluated against traditional QSM algorithms—including deep learning QSM, QUASAR (quantitative susceptibility and residual mapping), morphology‐enabled dipole inversion (MEDI), fast nonlinear susceptibility inversion (FANSI), and superfast dipole inversion (SDI)—using realistic digital brain models with and without microstructure effects, as well as in vivo human brain data. Quantitative assessments focused on susceptibility estimation accuracy, artifact reduction, and anatomical consistency.

**Results:**

In digital brain models, DEEPOLE outperformed conventional QSM methods by producing susceptibility maps with fewer artifacts and greater quantitative accuracy, especially in regions affected by microstructure effects. In vivo, DEEPOLE generated more anatomically consistent susceptibility maps and mitigated artifacts such as inhomogeneities and streaking, providing improved susceptibility estimates in deep gray matter and white matter.

**Conclusion:**

Incorporating macroscopically nondipolar Larmor frequency shifts into QSM through DEEPOLE improves the quality and accuracy of susceptibility maps. This methodological advancement enhances the reliability of susceptibility measurements, particularly in studies of neurodegenerative and demyelinating conditions where macroscopically nondipolar contributions are substantial.

## 1 INTRODUCTION

MRI phase data capture tiny local shifts in the proton Larmor frequency. These frequency shifts arise from tissue magnetization in the MR scanner's static magnetic field and relate to tissue content of paramagnetic iron and diamagnetic myelin.^1^ Various quantitative susceptibility mapping (QSM) algorithms have been proposed to translate phase data into maps of the underlying magnetic susceptibility distribution.^2^, ^3^, ^4^ QSM algorithms are increasingly being applied in clinical studies of neurodegeneration and iron homeostasis.^5^ However, in magnetically inhomogeneous tissues such as brain white matter, the physical model used for QSM is known to be simplistic.^6^ Simulations and preclinical in vivo studies have shown that QSM methods can substantially misestimate susceptibility.^6^, ^7^ In addition to inhomogeneity in white matter, the estimated susceptibility of the cerebrospinal fluid (CSF) in the ventricles often shows large variations. Moreover, the interfaces between ventricles and white matter are frequently inconsistent with the well‐defined boundaries seen on magnitude images.^6^

The prevailing physical model of QSM relies on strong assumptions about the tissue: isotropy, homogeneity, and that magnetic susceptibility is the exclusive source of phase shifts. These assumptions simplify the mathematical relationship between the microscopic scale magnetic tissue architecture and mesoscopic scale (voxel‐average) frequency measurements. Specifically, these assumptions permit modeling microscopic magnetism using the Lorentzian approach^8^ with a spherical cavity^2^, ^9^, ^10^ and neglect differential relaxation rates of tissue compartments.^11^ However, evidence suggests that these assumptions are inadequate for anisotropic, compartmentalized^12^, ^13^ brain tissues, particularly in highly myelinated white matter (WM).^11^, ^14^, ^15^, ^16^, ^17^, ^18^, ^19^, ^20^ The third assumption posits that magnetic susceptibility is the sole source of phase shifts. Experimental evidence challenges this, showing substantial voxel‐average Larmor frequency shifts caused by interactions between free water hydrogen nuclei and macromolecular protons in lipids and proteins.^21^ Assessing the magnitude of these error sources on susceptibility maps in vivo is challenging. This difficulty arises from the lack of a suitable in vivo gold standard for magnetic susceptibility and methods that can robustly incorporate physical models accounting for microstructure effects and chemical exchange.

In this work, we propose DEEPOLE (deep learning of the phase origin with a Lorentzian sphere estimation), a clinically applicable, physics‐informed deep learning method for QSM in magnetically inhomogeneous tissues. We evaluated the performance of our method against conventional QSM methods both in silico and in vivo.

## 2 METHODS

### 2.1 Quantitative susceptibility and residual mapping: An extended forward model for tissue‐induced Larmor frequency shifts

Assuming a homogeneous, structurally isotropic medium, magnetic susceptibility ***χ*** causes Larmor frequency shifts ***f***_***χ***_ that can be described through a three‐dimensional convolution (*) of ***χ*** with a unit magnetic dipole response, **d**, incorporating a Lorentz sphere correction and where ***d*(0)** = 0 (as detailed in Schweser et al.^2^):

(1) $$f_{\chi }=d*\chi .$$

Current single‐orientation QSM algorithms solve Eq. (1) using different deconvolution approaches (***χ = d*** *^−1^ ***f***_***χ***_). For media with anisotropic magnetic susceptibility described by a 3 × 3 susceptibility tensor, Eq. (1) only accounts for the frequency distribution caused by the *χ*_33_ component of the laboratory‐frame tensor—the apparent susceptibility. Frequency shifts resulting from the *χ*_13_ and *χ*_23_ elements follow convolution relations similar to Eq. (1) but use different convolution kernels.^22^

**FIGURE 1:**
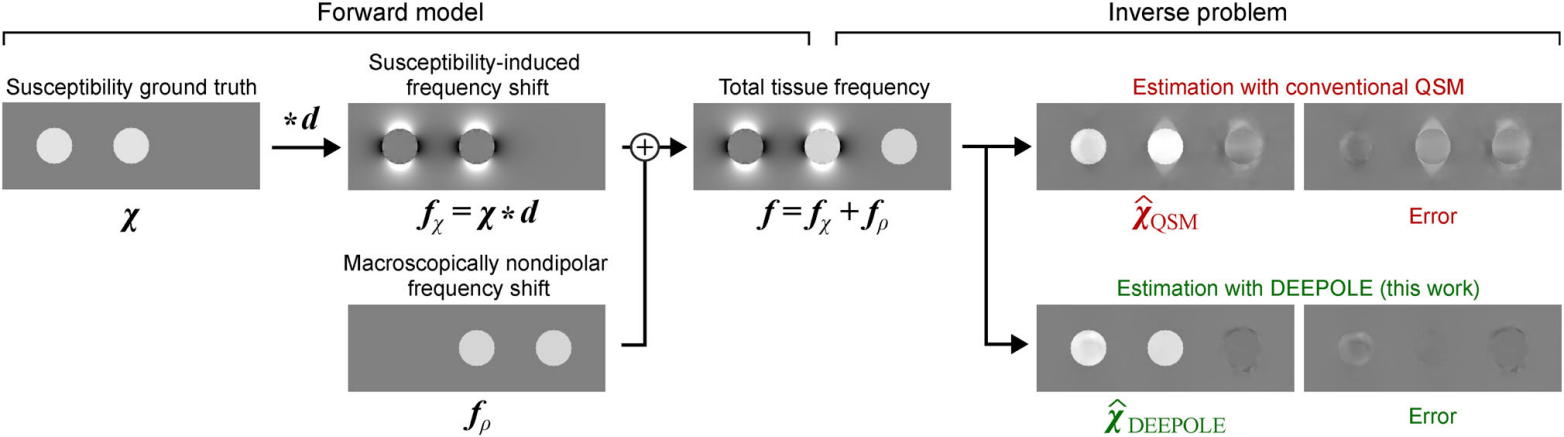
Simulation of three spherical sources to illustrate the forward model and inverse problem of susceptibility mapping with macroscopically nondipolar frequency contributions. Tissue structures can produce frequency contrast through magnetic susceptibility (*sphere on the left*), nondipolar frequency shift mechanisms (*sphere on the right*), or both (*sphere in the middle*). Magnetic susceptibility, ***χ***, leads to characteristic dipole‐like frequency shifts, ***f***_***χ***_. The observable frequency shift, ***f***, contains ***f***_***χ***_ as well as nondipolar frequency shifts, ***f***_***ρ***_. The inverse problem of estimating ***χ*** from ***f*** simultaneously (*right part of figure*) involves the source separation and the inversion of the dipole convolution. DEEPOLE, deep learning of the phase origin with a Lorentzian sphere estimation; QSM, quantitative susceptibility mapping.

If a QSM algorithm based on Eq. (1) is applied to a frequency map with local frequency shifts, it misattributes these shifts to susceptibility variations, which results in non‐local artifacts in ***χ***,^6^, ^7^ as illustrated in Figure 1 (right). A more detailed discussion of the nature of these artifacts may be found in the Appendix.

The quantitative susceptibility and residual mapping (QUASAR) model^6^ introduced an additive term ***f***_***ρ***_ for local frequency shifts to mitigate the non‐local artifacts resulting from Eq. (1), as follows:

(2) $$f=f_{\chi }+f_{\rho }=\left(d*\chi \right)+f_{\rho },$$

where ***f*** is the total observed frequency shift distribution (illustrated in Figure 1).

Generally, the term ***f***_***ρ***_ represents a composite of contrast mechanisms that do not follow the characteristic dipole pattern described by Eq. (1). This includes non‐local shifts from *χ*_13_ and *χ*_23_, echo time–dependent voxel‐average frequency shifts related to multi‐compartment effects,^11^, ^12^, ^23^, ^24^ and imaging artifacts like head movements or Gibbs ringing. We refer to ***f***_***ρ***_ as macroscopically nondipolar frequency shifts to denote their deviation from the macroscopically dipolar pattern described by Eq. (1). For brevity, “macroscopically” is occasionally omitted in the present work. Note that while these ***f***_***ρ***_ shifts may arise from microscopically dipolar interactions, they do not produce the characteristic macroscopically dipolar field pattern.

Estimating ***χ*** from ***f*** requires a priori knowledge of ***f***_***ρ***_, ***χ***, or both, because the inverse problem is underdetermined. Without additional constraints, a trivial solution to Eq. (2) is ***f***_***ρ***_ = ***f*** and ***χ*** = 0. Schweser et al.^25^ addressed Eq. (2) by restricting ***f***_***ρ***_ to local phase effects related to the generalized Lorentzian approximation^14^ for parallel fibers. The model used fiber directions from additionally acquired diffusion data and in vivo brain field maps acquired at 3 T with the head in different orientations relative to the main magnetic field. Sandgaard et al.^20^, ^26^ recently extended this approach to account for orientation dispersion. They demonstrated the solution in an ex situ mouse brain at 16.4 T using a single‐orientation field map and multishell diffusion data.^19^ The original QUASAR publication attempted to solve Eq. (2) without strong assumptions about the underlying signal model or the need for additional MRI scans. They used an iterative algorithm with a piece‐wise constant constraint for ***f***_***ρ***_ in the cerebrospinal fluid (CSF), Tikhonov regularization, and a scale parameter for ***f***_***ρ***_. However, the method was only partially effective in solving Eq. (2), and the authors observed strong dependencies on algorithmic parameters.^6^

### 2.2 Deep learning–based susceptibility mapping using the QUASAR model

**FIGURE 2:**
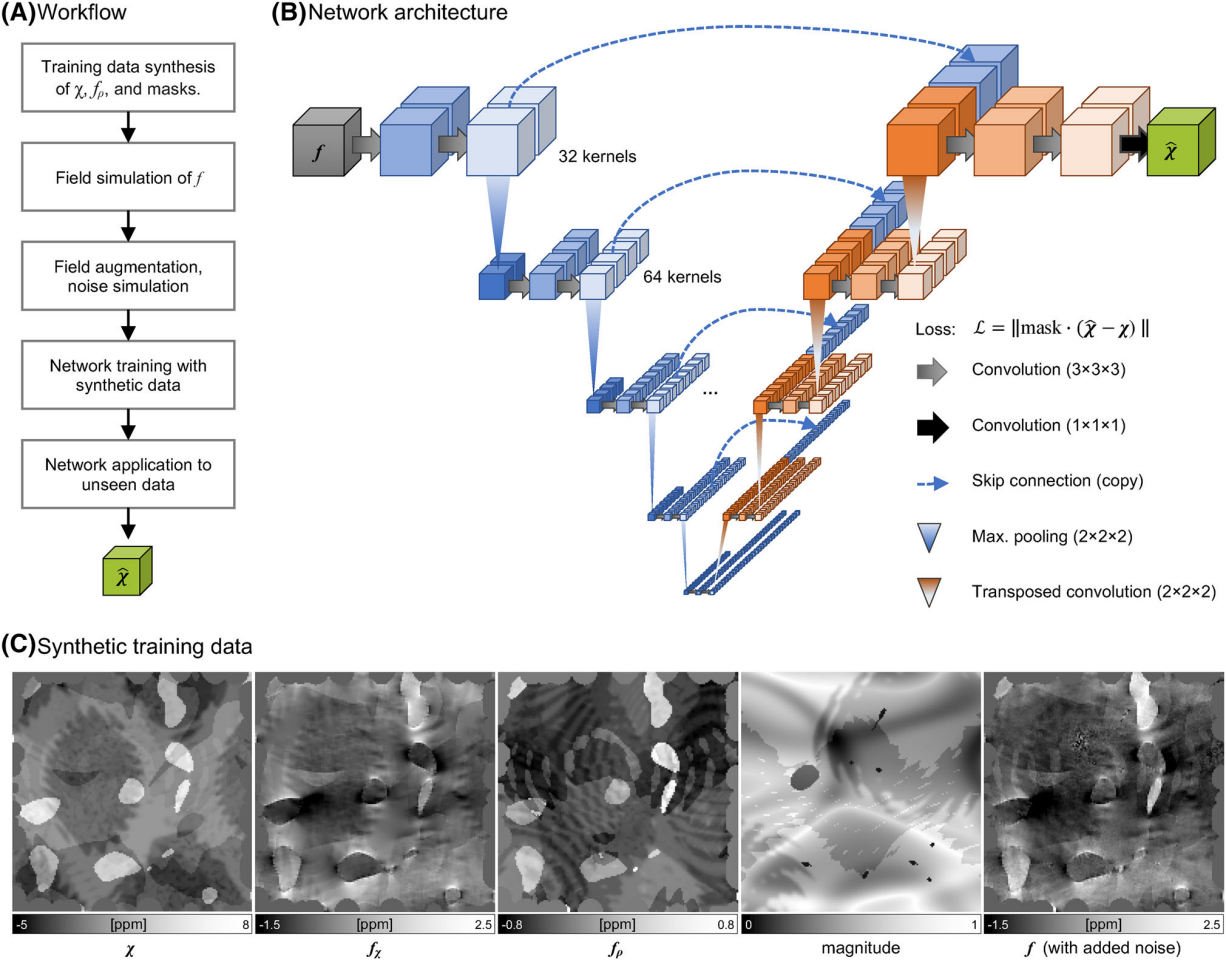
Deep learning of the phase origin with a Lorentzian sphere estimation (DEEPOLE) workflow and U‐Net neural network architecture. (A) Steps of the DEEPOLE approach to provide susceptibility estimates. (B) Our implementation of the three‐dimensional U‐Net neural network architecture. During training, the differences between ground truth and estimate are masked before calculating the estimation error and loss. (C) Example of one set of synthetic training data, consisting of (*from left to right*) a susceptibility distribution, the corresponding simulated frequency shift, a nondipolar frequency perturbation, a magnitude, and the total frequency shift with magnitude‐dependent noise. The patterns feature different organic shapes, textures, and intensities. Low (*dark*) regions in the magnitude lead to high noise levels in the frequency map.

We propose using deep convolutional neural networks for solving the QUASAR model (overview in Figure 2A). This approach is motivated by the favorable abilities of convolutional neural networks in inverse imaging problems^27^ and, in particular, their successful previous application in attenuating streaking artifacts in the solution of Eq. (1).^28^, ^29^, ^30^ Conventional optimization methods are often forced to use simple regularization terms due to computational limitations. Deep learning methods, instead, can implicitly learn a domain‐adapted regularizer optimized for the specific problem represented in the training data.^27^, ^31^ Convolutional neural networks can be trained to learn an implicitly regularized inverse function mapping ***f*** to ***χ*** using examples of input (***f***) and output volumes (***χ***).^32^, ^33^

Similar to Bollmann et al.,^29^ we generated pseudo‐random synthetic data to train a neural network in the absence of in vivo ground‐truth data and used the U‐Net architecture^34^ for its proven effectiveness in image‐to‐image translation tasks (Figure 2B). The network was implemented using Keras/TensorFlow library version 2.3.0.^35^, ^36^ We used Eq. (2) to synthesize training data adhering to the QUASAR model and a custom pattern generator, yielding images with power spectral densities similar to those of natural images (see Appendix). We describe further details on the synthesis of the training data and the training process in the Appendix.

We posited that physically meaningful samples of ***χ*** and ***f***_***ρ***_ should resemble the power spectral densities of natural images. In particular, the power spectrum of ***f***_***ρ***_ should not exhibit the characteristic anisotropic energy distribution imprinted by the dipole convolution in Eq. (1). We hypothesized that neural networks would produce solutions for Eq. (2) matching the expected power spectra distributions, provided the training data exhibited these patterns. We chose to use pseudo‐random synthetic patterns compared with anatomical images, as proposed in another study,^28^ to ensure that the network learns a generic mathematical solution to the inverse physics problem rather than memorize typical anatomical patches or patterns.

### 2.3 QSM algorithms

We used the same pipeline used for training DEEPOLE, along with the same random patterns of ***χ***, to train a network based on the standard QSM model in Eq. (1). We refer to this sibling method, which is similar to the method proposed by Bollmann et al.,^29^ as deep learning QSM (DL QSM).

In addition to DL QSM, we selected the following QSM algorithms for comparison with DEEPOLE: (1) QUASAR,^6^ the original proof‐of‐concept algorithm for solving Eq. (2), with parameters optimized as in the original publication; (2) (morphology‐enabled dipole inversion [MEDI], toolbox version 2020‐01‐15),^37^ a widely recognized and applied QSM algorithm; (3) fast nonlinear susceptibility inversion (FANSI), (FANSI toolbox v2.0, 2020‐07‐27),^38^ a top‐scoring algorithm in the recent QSM reconstruction challenge^3^ (normalized root mean square error at Stage 2); and (4) superfast dipole inversion (SDI),^39^ a direct filtering algorithm that solves the inversion problem without iterative refinements or spatial‐domain regularization.

### 2.4 Validation in a digital brain model

**FIGURE 3:**
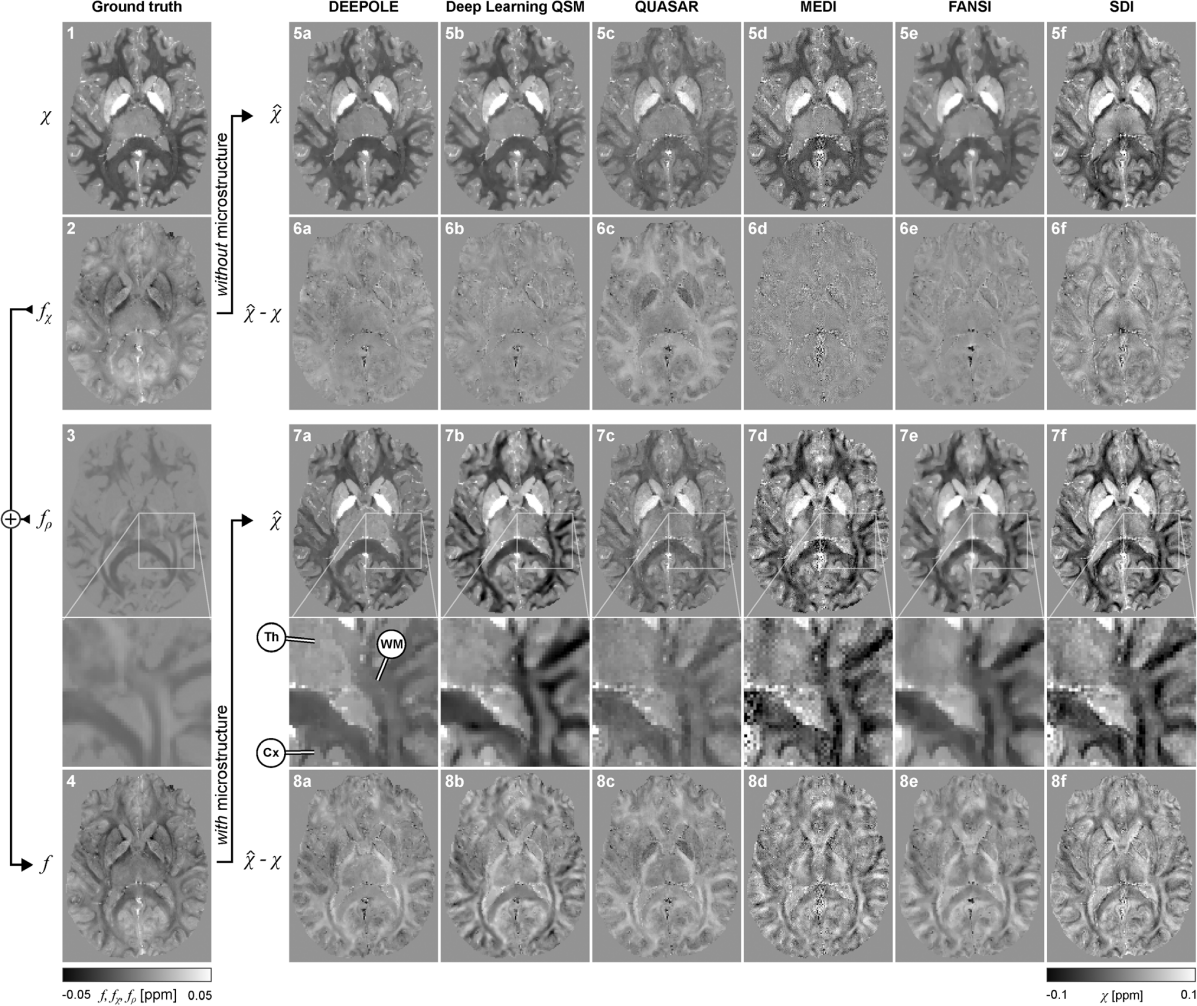
Comparison of susceptibility maps computed with different algorithms in a realistic digital brain. Tiles 1–4 show axial slices of the digital brain (ground truth). Susceptibility was estimated from the purely susceptibility‐induced frequency (2) and from the frequency map that included macroscopically nondipolar contributions (4). Markers in the enlarged insets indicate cortical boundaries (Cx), white matter (WM), and thalamus (Th). DEEPOLE, deep learning of the phase origin with a Lorentzian sphere estimation; FANSI, fast nonlinear susceptibility inversion; MEDI, morphology‐enabled dipole inversion; QSM, quantitative susceptibility mapping; QUASAR, quantitative susceptibility and residual mapping; SDI, superfast dipole inversion.

To validate our inversion technique against a ground truth and compare it with other QSM algorithms, we created two digital models. One model included microstructure effects, and the other did not. We built the models based on the realistic digital brain model of the 2019 QSM Challenge (*Sim2*; signal‐to‐noise ratio = 100).^40^ Using a realistic brain model ensured that the evaluation closely mimicked in vivo conditions. It incorporated image features not present in the synthetic training data, including realistic tissue interfaces, textures, and k‐space sampling artifacts.^40^ The original model did not contain microstructure effects. To create the model with microstructure effects, we added macroscopically nondipolar frequency contributions to the white matter (Figure 3, panel 3). These were derived from diffusion images according to Wharton and Bowtell,^7^ as proposed by the authors of the original challenge data set.^40^ Details may be found in the Appendix.

We quantified overestimation or underestimation of the susceptibility estimates in deep gray matter (DGM) with a least‐squares linear fit (*SciPy* version 1.13.1)^41^ that compared the estimated susceptibility mean values with the ground‐truth values (***χ***_estimate_ = slope · ***χ***_ground truth_ + offset) across six DGM regions (caudate, globus pallidus, putamen, red nucleus, dentate nucleus, thalamus). A slope value close to unity indicates high quantitative accuracy, independent of the reference. We quantified the visual similarity between the ground truth and computed susceptibility maps using the XSIM metric^42^—a structural similarity index adapted for susceptibility maps. Additionally, following the approach of the QSM Challenge 2.0 report,^3^ we calculated the demeaned normalized root mean squared error. This metric was highly correlated with all other global metrics compared in the report. All metrics were calculated exclusively from voxels within the brain. For the region‐wise analyses, we used the region of interest (ROI) masks provided in the data set and eroded each region by one voxel to reduce partial volume errors. Due to resimulation of the challenge model and adjustments to regularization parameters for the QSM algorithms, slight differences from the official challenge report results were expected.

### 2.5 In vivo assessment

To investigate the effect of macroscopically nondipolar frequency shifts on in vivo susceptibility estimates, we compared the different algorithms using data from a heterogenous cohort of 8 subjects. The cohort included 1 healthy volunteer and 7 people with multiple sclerosis (age range 24–58 years, 3 males, 5 females), representing a range of magnetic susceptibilities and structural variations. The local Ethical Standards Committee approved the human experiments, and written informed consent form was obtained from all participants. Data were acquired at 3 T (Philips MR7700) with a 32‐channel brain coil using a monopolar three‐dimensional multi‐echo gradient‐recalled echo sequence for phase and magnitude images (transverse anterior commissure–posterior commissure; field of view = 256 × 184 × 144 mm^3^, matrix size = 512 × 512 × 144, nominal resolution = 0.5 × 0.5 × 1 mm^3^, repetition time [TR]/echo time [TE]/ΔTE = 50 ms/3.7 ms/6 ms, number of echoes = 8, flip angle = 15°, bandwidth = 283.9 kHz, compressed‐sensing factor = 5, acquisition time = 7 min 0 s) and a magnetization‐prepared turbo field echo sequence for T_1_‐weighted imaging (sagittal anterior commissure–posterior commissure; field of view = 257 ×257 × 180 mm^3^, matrix size = 352 × 352 × 240, nominal resolution = 0.73 × 0.73 × 0.75 mm^3^, TR/TE = 9.63 ms/4.525 ms, flip angle = 8°, bandwidth = 158 kHz, compressed‐sensing factor = 5, acquisition time = 4 min 50 s). Phase images, reconstructed by the scanner, were unwrapped using a best‐path algorithm.^43^ Background fields were removed using variable‐radius SHARP.^44^, ^45^ For DEEPOLE and DL QSM, the field maps were spatially resampled to isotropic voxel aspect ratios using mri_convert from *FreeSurfer* ^46^ with cubic interpolation. They were then rotated to axial orientation using FSL‐FLIRT^47^ with trilinear interpolation. For the quantitative analyses, we used 1.0‐mm voxel edge length to maintain consistency with the digital brain model data. For visual display, we retained the native in‐plane resolution of 0.5 mm to preserve fine‐scale features. Computed susceptibility maps were rotated back to their original orientation, resampled to the original voxel size, and referenced to the whole brain.

We estimated mean susceptibilities in multiple ROIs. To create CSF and DGM ROIs (caudate, globus pallidus, putamen, red nucleus, dentate nucleus, thalamus, substantia nigra), we used a bi‐parametric^48^, ^49^ (QSM and T_1_‐weighted) multi‐atlas segmentation technique optimized for QSM, bi‐parametric Joined Label Fusion (https://gitlab.com/R01NS114227/antsjointlabelfusion_biparametric).^48^ To create WM ROIs, we used *SynthSeg* ^50^ on the T_1_‐weighted templates. We propagated the ROIs to native subject spaces using bimodal warp fields from ANTs^51^ nonlinear registrations. In native space, we eroded each region by one voxel to reduce errors from partial volumes.

Similar to the ground‐truth comparison in the brain model, we quantified the relative overestimation or underestimation between the DGM susceptibility estimates from the different methods using a linear model (***χ***_method Y_ = slope · ***χ***_method X_ + offset), where methods X and Y represent the algorithms being compared. We fitted one model for each subject and report the mean slope across all subjects. Unlike the brain model with known ground truth, both the dependent and the independent variables in our in vivo data contain measurement errors. Therefore, instead of least‐squares linear regression, we used pairwise orthogonal distance regression (*SciPy* version 1.13.1).^41^

## 3 RESULTS

### 3.1 Digital brain model

**FIGURE 4:**
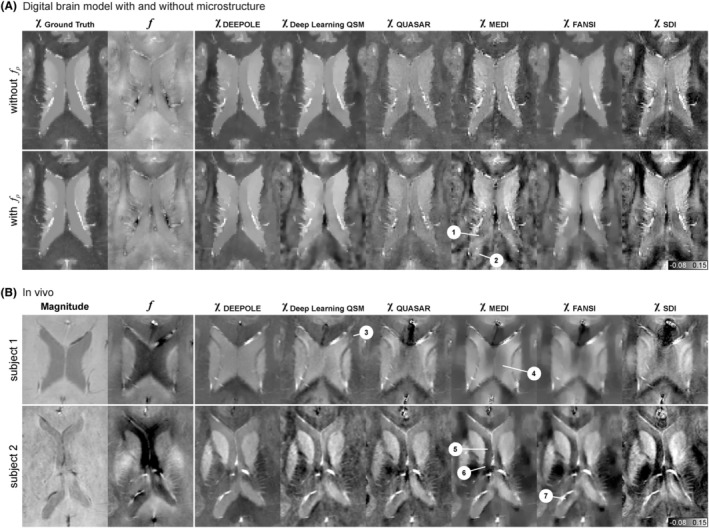
Comparison of homogeneity and anatomical boundaries in the estimates from different algorithms. (A) Enlarged axial cross sections through the lateral ventricles of the digital brain models show underestimation (*Marker 1*) and inconsistent tissue boundaries (*Marker 2*) in the quantitative susceptibility mapping (QSM) methods after addition of microstructure to the simulation (*second row*). DEEPOLE (deep learning of the phase origin with a Lorentzian sphere estimation) and QUASAR (quantitative susceptibility and residual mapping) were less affected by the nondipolar perturbations. (B) Similar to the simulation, the in vivo cases (Subject 1: male, 38 years, normal brain; Subject 2: female with multiple sclerosis, 52 years) present with inconsistent tissue boundaries (*Markers 3 and 6*) and inhomogeneity (*Markers 3, 4, 5, and 7*) in the QSM methods. The QUASAR method had higher inhomogeneity in vivo than in the simulation. The susceptibility estimates from DEEPOLE have homogenous intensity and the tissue boundaries are consistent with the magnitude images (*left*). FANSI, fast nonlinear susceptibility inversion; MEDI, morphology‐enabled dipole inversion; SDI, superfast dipole inversion.

Figure 3 illustrates the simulated digital brain model, the estimated susceptibility maps from the different algorithms, and their differences to the ground truth. In the case without microstructure, all methods except QUASAR and SDI (5a,b,d,e) showed only minor deviations (6a,b,d,e) from the ground truth (1). QUASAR produced well‐defined tissue boundaries and homogeneous regions but significantly underestimated the susceptibility. SDI was the most affected by artifacts among all methods, leading to less reliable susceptibility maps. When microstructure effects were added, all methods (7a–f) demonstrated substantial deviations (8a–f) from the ground truth. To varying degree, all methods misinterpreted the macroscopically nondipolar contributions (3) as susceptibility. This led to weak definitions of cortical (Cx in 7a) boundaries and strong inhomogeneities in the WM, which was relatively homogeneous in the ground truth, with DEEPOLE being the least affected (8a). Discrepancies were especially pronounced in regions with strong nondipolar contrast, such as the optic radiations and the corpus callosum. As expected, introducing nondipolar effects in WM led to substantial errors in areas without simulated nondipolar contributions. These errors manifested as underestimation, inhomogeneities, and artifactual boundaries, particularly in the lateral ventricles (Figure 4A). Among all methods, DEEPOLE produced the least corrupted susceptibility maps overall.

**FIGURE 5:**
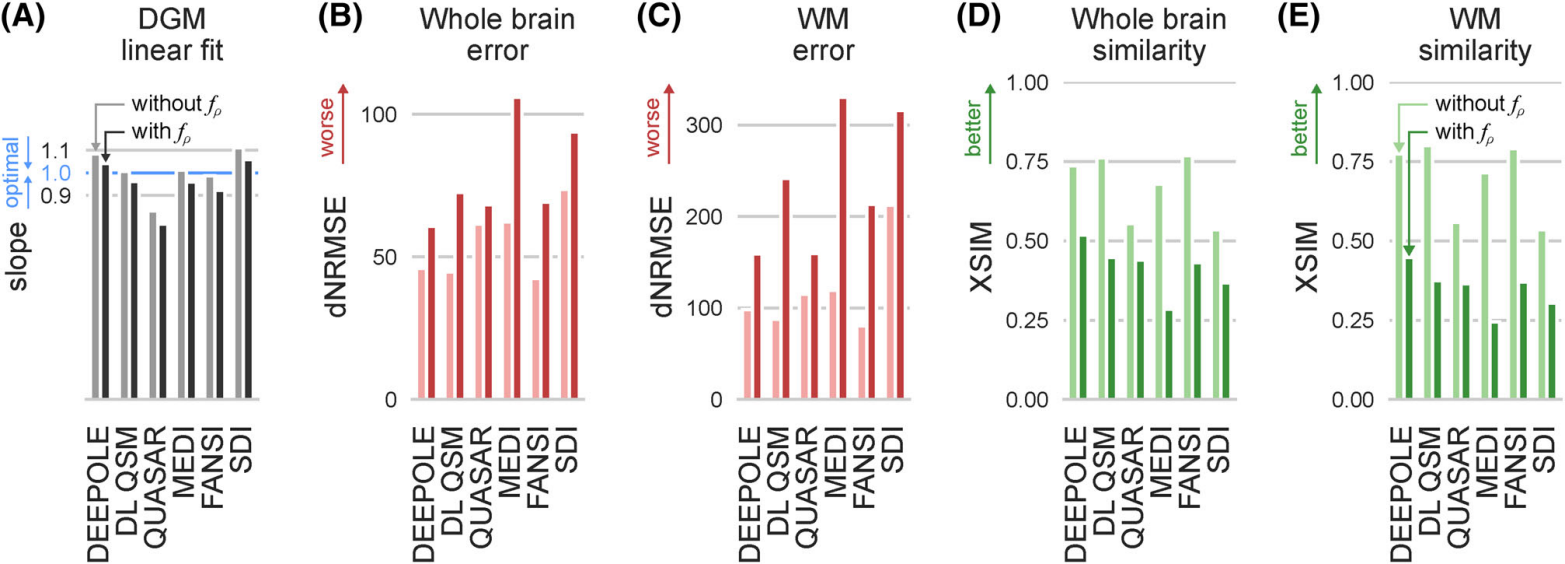
Linear regression slope (A), demeaned normalized root mean square error (B,C), and structural similarity index (D,E) between the digital brain ground truth and the susceptibility estimates from different algorithms. Light bars show the results from the simulation *without* microstructure, and dark bars show the results *with* microstructure in the white matter. Slope values larger than 1 indicate overestimation and smaller than 1 underestimation (A). Without microstructure, all algorithms except for SDI gave comparable results. With microstructure, solutions from all algorithms were degraded. DEEPOLE (deep learning of the phase origin with a Lorentzian sphere estimation) was least affected by microstructure effects. DGM, deep gray matter; DL QSM, deep learning quantitative susceptibility mapping; dNRMSE, demeaned normalized mean square error; FANSI, fast nonlinear susceptibility inversion; MEDI, morphology‐enabled dipole inversion; QUASAR, quantitative susceptibility and residual mapping; SDI, superfast dipole inversion; WM, white matter.

Figure 5 summarizes the quantitative evaluation, which largely confirmed qualitative findings. Although all metrics degraded to varying degree between algorithms when microstructure effects were added (light bars are without microstructure, dark bars are with microstructure), DEEPOLE outperformed QSM methods in all metrics. In particular, all QSM algorithms except SDI resulted in susceptibility underestimation, whereas DEEPOLE maintained quantitative accuracy.

### 3.2 In vivo

**FIGURE 6:**
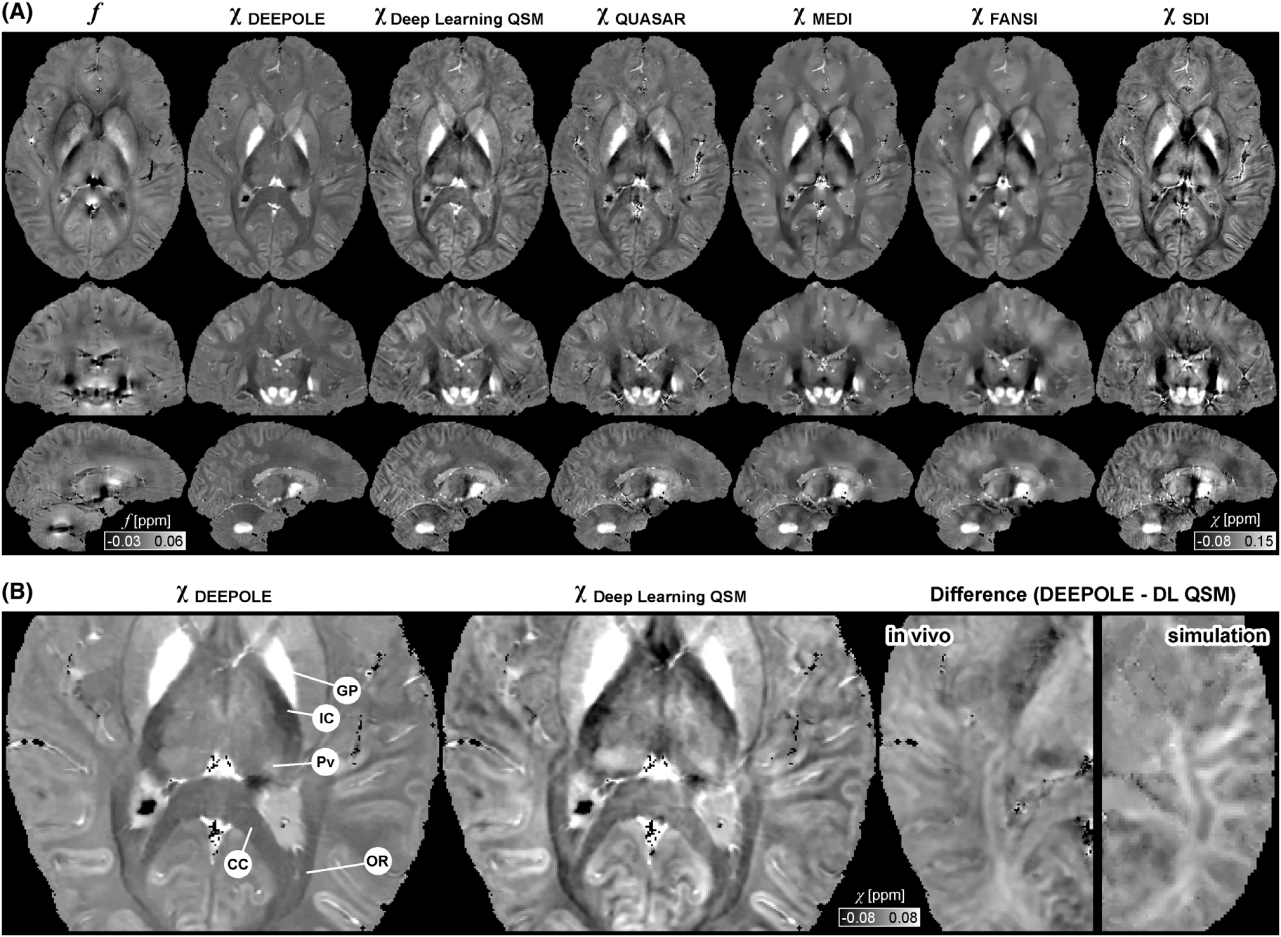
Comparison of susceptibility estimates from different algorithms in a representative subject (female with multiple sclerosis, 43 years). (A) Axial, coronal, and sagittal cross sections. (B) Enlarged axial cross sections of the susceptibility estimates from DEEPOLE (deep learning of the phase origin with a Lorentzian sphere estimation) and deep learning quantitative susceptibility mapping (DL QSM), and their difference, next to the corresponding difference image from the digital brain model (“Simulation”). Markers indicate optic radiations (OR), internal capsule (IC), corpus callosum (CC), globus pallidus (GP) and the pulvinar (Pv). FANSI, fast nonlinear susceptibility inversion; MEDI, morphology‐enabled dipole inversion; QUASAR, quantitative susceptibility and residual mapping; SDI, superfast dipole inversion.

Observations in the in vivo data, illustrated in Figures 4B and 6, closely mirrored the findings from the digital brain model with microstructure. DEEPOLE maps demonstrated the most uniform susceptibility throughout anatomical regions, with lower standard deviations compared with DL QSM (e.g., WM: standard deviation 12.6 vs. 20.2 ppb for the DL QSM, *p* < 0.001, one‐sided t‐test; CSF: standard deviation 26.2 vs. 36.1 ppb for the DL QSM, *p* < 0.001). Additionally, DEEPOLE provided the clearest definition of tissue boundaries among all methods, particularly in the coronal and sagittal views, whereas other maps showed cloud‐like susceptibility inhomogeneities and streaking artifacts. DEEPOLE substantially reduced anatomically incorrect signal variations, such as hypointensities in the lateral ventricles (Figure 4B), and artifactual tissue boundaries commonly observed in susceptibility maps. A direct comparison of DEEPOLE with DL QSM (Figure 6B) revealed that DEEPOLE had lower susceptibility values in the globus pallidus and pulvinar, and reduced contrast between gray matter (GM) and dense WM tracts, such as the optic radiations and corpus callosum (markers in Figure 6B). The in vivo difference images also showed a pronounced contrast between WM and cortical GM, highlighting differences in susceptibility estimation between the methods. We compared the observed in vivo differences between DEEPOLE and DL QSM with those from the digital brain model with microstructure (Figure 6B, right‐most panel). This comparison aimed to assess whether the effect of macroscopically nondipolar contributions was comparable between the real data and the simulation. Although we observed high contrast similarity in the WM and cortical regions, substantial deviations were seen in the DGM, particularly in globus pallidus and pulvinar.

**FIGURE 7:**
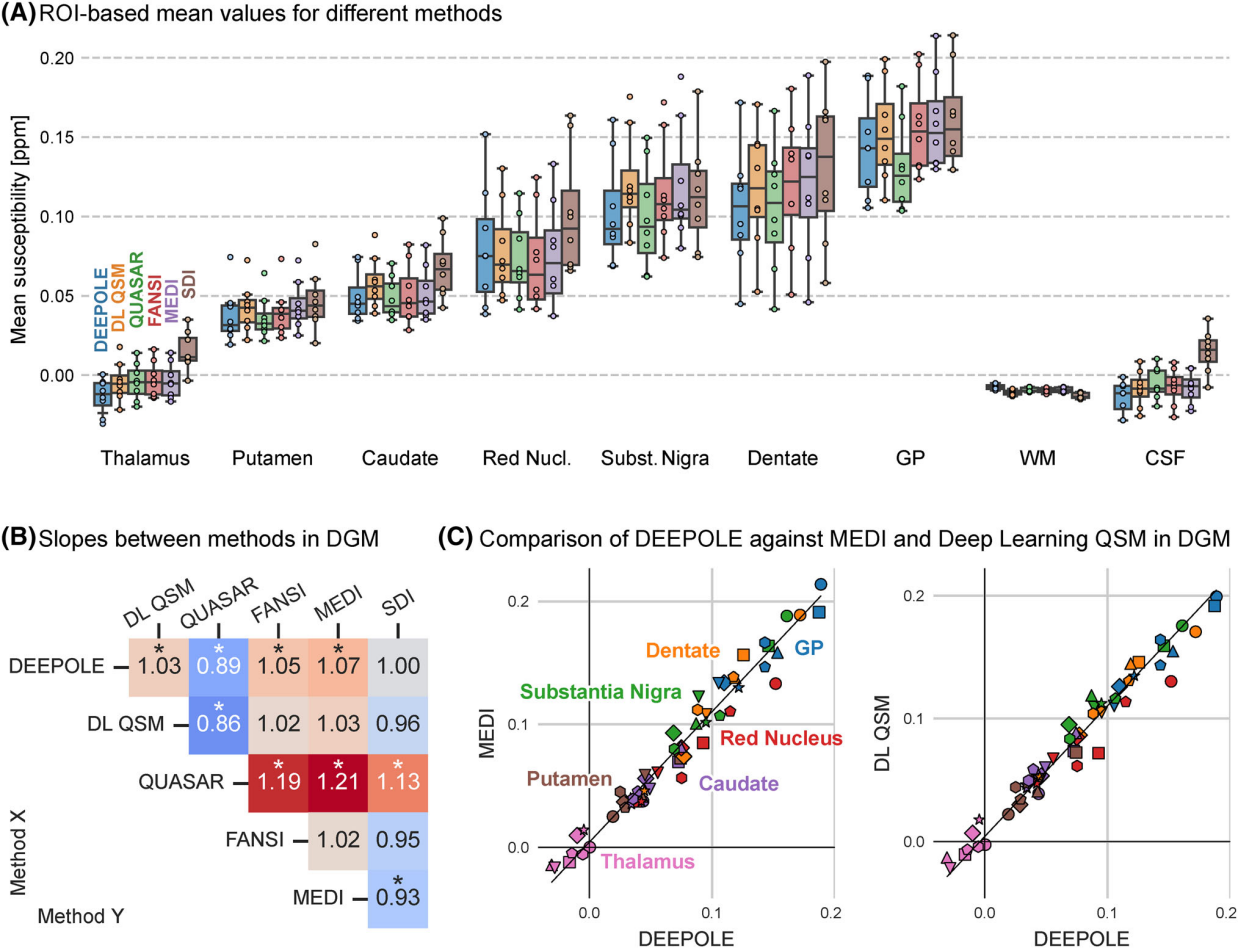
Comparison of in vivo magnetic susceptibility estimates from different algorithms. (A) Regional estimates. The boxes denote the quartiles of the magnetic susceptibility over the 8 subjects, and the whiskers span from the minimal to the maximal value. (B) Heatmap of mean slopes of orthogonal distance regressions between the mean region‐of‐interest (ROI) susceptibility estimates in deep gray matter (DGM). Each cell represents the mean of the slopes of the lines fitted across the values from the different ROIs and subjects for the respective pair of methods. Asterisks denote significant deviation from 1 (*p* < 0.05, one‐sample t‐test). (C) Comparison of individual ROI mean values from DEEPOLE (deep learning of the phase origin with a Lorentzian sphere estimation), DL QSM (deep learning quantitative susceptibility mapping), and MEDI (morphology‐enabled dipole inversion). Marker shapes indicate different subjects. CSF, cerebrospinal fluid; GP, globus pallidus; FANSI, fast nonlinear susceptibility inversion; QUASAR, quantitative susceptibility and residual mapping; SDI, superfast dipole inversion; WM, white matter.

Figure 7A summarizes the quantitative results. In most DGM structures, whole brain–referenced susceptibility estimates from DEEPOLE and QUASAR were slightly lower by 3%–7% compared with those from other methods (*p* < 0.05) and comparable to SDI. Pair‐wise correlations (Figure 7C) did not reveal region‐specific differences within the DGM, suggesting that the susceptibility differences were consistent across regions.

## 4 DISCUSSION

This study introduced DEEPOLE, a deep learning–based approach integrating the QUASAR model to account for frequency contributions not described by the conventional QSM model. DEEPOLE mitigated several substantial issues of conventional QSM algorithms, such as overly inhomogeneous susceptibility in WM and CSF. It also reduced artifactual boundaries between WM and ventricles that were inconsistent with magnitude images in vivo or the ground truth in silico. Because DEEPOLE relies on the same phase images as conventional QSM and does not require additional pulse sequences, it is suitable as a direct replacement for QSM in prospective and retrospective studies of the brain and the body. DEEPOLE runs in seconds on state‐of‐the‐art CPUs, whereas some iterative QSM methods require hours of computation. Therefore, DEEPOLE can be applied efficiently to large cohorts and in the clinical setting.

### 4.1 Effect of macroscopically nondipolar Larmor frequency shifts on susceptibility maps

It is well established that conventional QSM methods require regularization strategies to manage the amplification of measurement noise in the susceptibility map. In the presence of macroscopically nondipolar frequency contributions, a second purpose of regularization emerges: suppression of streaking artifacts caused by deconvolving the nondipolar contributions with the dipole function (Eq. [1]; Appendix). The way nondipolar frequencies manifest on susceptibility maps depends on the regularization strategy and, hence, can differ substantially between algorithms, which was also observed in our study. Consistent with the findings from the 2016 QSM challenge,^52^ normalized root mean square error values, which were strongly correlated with other global metrics in a previous study,^3^ did not reflect the subjectively perceived differences in the visual quality between the algorithms.

By assessing modern QSM algorithms, using more realistic brain models, and providing quantitative analyses, our work complemented earlier observations of macroscopically nondipolar frequency shifts by Wharton and Bowtell^7^ in humans and Schweser et al.^6^ in mice. For example, Schweser et al.^6^ (Figure 4 in their publication) demonstrated in mice that a nondipolar frequency difference between the CSF and brain tissue was necessary to properly reconstruct susceptibility maps consistent with the underlying anatomy, which we observed similarly in the human brain.

Although DEEPOLE did not entirely resolve artifacts related to nondipolar frequency shifts, it was more effective at suppressing them than the tested QSM algorithms. In a preliminary analysis using in vivo multi‐orientation data, we also found that DEEPOLE reduced the orientation dependency of the calculated susceptibility compared with DL QSM.^53^ This observation may be explained by orientation‐dependent nondipolar frequency shifts misinterpreted as susceptibility by conventional QSM method. Future research will study these effects and determine whether residual artifacts on the DEEPOLE susceptibility maps can be reduced through improvements in the network architecture and training data, or if additional information and more tissue‐specific biophysical modeling is required.

### 4.2 In vivo validation

An independent in vivo demonstration of macroscopically nondipolar frequency effects is still lacking due to the absence of methods to isolate these effects. However, the finding that similarly algorithm‐specific regularization artifacts were seen on susceptibility maps in vivo (Figure 6) and in the brain model with microstructure (Figure 3)—but not in the model without microstructure—provide additional corroborative support for the presence of significant nondipolar effects in vivo.

### 4.3 Discrepancies between in vivo and digital model

Discrepancies between in vivo findings and those in digital models were most prevalent in the DGM (Figure 6B). These discrepancies suggest that our simulation did not properly model the qualitative features of in vivo nondipolar contributions. A reason for discrepancies in the DGM may be that nondipolar contrast is not restricted to the WM, as simulated in our model. Nondipolar contrast not simulated in our brain model but present in vivo may be related to diffusion exchange effects^54^ and chemical exchange,^21^, ^55^ and modulated by varying types and concentrations of proteins and lipids. However, because previous observations on the magnitude of protein‐induced frequency shift in brain tissue were inconsistent,^21^, ^56^, ^57^, ^58^ we refrained from including these effects in the brain model.

The observed discrepancy in the DGM susceptibility estimations between conventional QSM methods and DEEPOLE may partly be related to such effects. By comparing the slopes of the linear fits between the DGM mean values, we found that QSM methods underestimated susceptibility in the model with microstructure, whereas DEEPOLE estimated the values in this scenario more accurately. Conversely, we found that QSM methods yielded higher susceptibility compared with DEEPOLE in vivo. If results in the digital model generalize to the real world (i.e., susceptibility values from DEEPOLE are quantitatively more correct than those from QSM), our in vivo results suggest that conventional QSM may overestimate DGM susceptibility in vivo. Future research on frequency contrast mechanisms in the DGM is needed to test these hypotheses.

### 4.4 Similar approaches

Chen et al.^59^ recently proposed accounting for microstructure‐related frequency effects in QSM through the hollow‐cylinder model of axons.^11^ Specifically, they determined microstructure‐related contributions based on the well‐established nonlinearity of the phase evolution with T_E_, resulting from differences in compartmental relaxation times.^11^, ^60^ Although the authors demonstrated microstructure phase contrast similar to that known from frequency difference mapping applications,^23^ the effect of the correction on their susceptibility estimates was minimal. The key conceptual distinction between this method and DEEPOLE is that DEEPOLE does not restrict macroscopically nondipolar contributions to multicompartment relaxation effects (which are nonlinear with T_E_). Instead, DEEPOLE also accounts for time‐invariant effects, such as chemical exchange and field perturbation related to off‐diagonal elements of the susceptibility tensor.^22^ Although Chen et al. included chemical shift effects within the myelin compartment of their hollow cylinder model, this approach is insufficient for describing similar effects occurring outside myelin, such as those related to extraneuronal proteins. The comparatively greater improvement in susceptibility maps achieved with DEEPOLE further supports the hypothesis that significant chemical exchange or other time‐invariant frequency effects exist in the brain.

Feng et al.^61^ recently extended susceptibility tensor imaging^62^ by incorporating an orientation‐independent nondipolar frequency shift term. Although the modification improved reconstruction quality, the authors noted that it did not account for orientation‐dependent nondipolar effects, which the method would still erroneously assign to susceptibility tensor elements. Because DEEPOLE operates on single‐orientation data, its mathematical treatment of orientation‐dependent and orientation‐independent nondipolar effects is identical, inherently accommodating orientation‐dependent effects.

### 4.5 Practical implications

The observed shift of DGM susceptibility values obtained with DEEPOLE compared with conventional QSM methods in vivo (Figure 7A), even though the slope was close to 1, may be attributed to a reduction in the susceptibility estimates in the reference region. We used the whole brain as a reference. WM is the tissue type with the largest volume fraction of the whole brain (24% in this study's cohort), and WM was more diamagnetic on DEEPOLE susceptibility maps (Figure 6). These considerations raise a critical concern regarding referencing in QSM clinical research: If QSM artifacts from nondipolar contributions of the reference region preferentially affect one of the studied groups, these artifacts may lead to false‐positive group differences with conventional QSM. Axonal damage and myelin disintegration, which occur in many neurological disorders, have been shown to cause a reduction in the microstructure‐related macroscopically nondipolar frequency shifts and those from chemical exchange.^18^, ^63^ This may result in biased QSM study outcomes in neurodegenerative and demyelinating conditions that involve whole‐brain or WM referencing. Our results suggest that macroscopically nondipolar effects increase the apparent average WM susceptibility in the human brain, consistent with findings in a fixed ex vivo mouse brain.^19^ In studies comparing a neurodegenerative condition to a control condition, this effect would lead to a stronger artificial reduction of referenced susceptibility values in the control group. Consequently, this would bias the study outcome toward increased susceptibility in the patient group. Increased susceptibility is a frequent finding in neurodegenerative conditions.^64^ Future research will have to determine to what degree such bias exists and may be mitigated by DEEPOLE.

## 5 CONCLUSION

By incorporating macroscopically nondipolar contributions into the susceptibility computation, DEEPOLE produced susceptibility maps that were quantitatively more accurate and exhibited fewer artifacts than conventional QSM methods. This improvement has the potential to reduce bias in QSM studies of conditions that differentially affect nondipolar contributions, thereby enhancing the reliability of susceptibility measurements in neurodegenerative and demyelinating diseases.

## CONFLICT OF INTEREST

All authors declare they have no competing interests.

## Figures and Tables

**FIGURE A.1:**
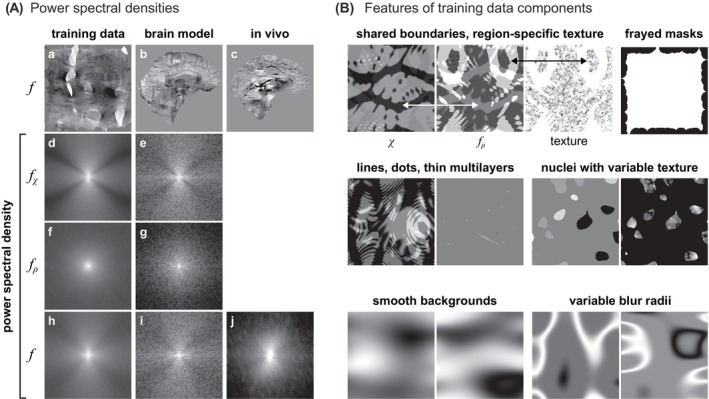
(A,B) Mean power spectral densities (PSDs; A) and features of the synthetic training data (B). (A) The PSDs from the simulated ***f***_***χ***_ (d,e) show a cone pattern, related to the Fourier‐transformed magnetic dipole kernel. The PSDs from the simulated ***f***_***ρ***_ are isotropic. The PSDs from the simulated ***f*** (h,i) resemble the PSD from in vivo ***f*** (j). The PSDs from the training data were averaged over all 1200 training samples and from the in vivo data over all 8 subjects, leading to different noise levels in the depicted PSD maps. (B) The synthetic training data are assembled from multiple components, mimicking different image properties of medical images. The samples of ***χ*** and ***f***_***ρ***_ are generated in pairs where some of the random structures in ***χ*** and ***f***_***ρ***_ share boundaries.

## Data Availability

Source code to generate the training data and to train the neural network are publicly archived in a Zenodo repository at https://doi.org/10.5281/zenodo.6806390. The digital brain models are archived at https://doi.org/10.5281/zenodo.14969113. The source codes of the compared third‐party QSM methods should be retrieved from their respective authors or their official repositories. The imaging data supporting the findings of this study are available from the corresponding author upon reasonable request and subject to institutional review board approval; these data are not publicly accessible due to privacy and ethical considerations.

## A.1. Nonlocal artifacts in conventional QSM related to macroscopically nondipolar frequency contrast

The dipole kernel, **d**, has a zero value at the origin (***d*[O]** = 0), which is essential for the mathematically simple convolution in Eq. (1). Writing the problem in this form is key to enabling numerical solutions using inverse filtering or iterative optimization methods. The origin constraint implies that the average susceptibility‐induced frequency shift in a voxel, ***f***_***χ***_(*i,j,k*), is determined entirely by non‐local field shifts from surrounding susceptibility distributions. The susceptibility of the voxel itself, ***χ*** (*i,j,k*), does not affect ***f***_***χ***_(*i,j,k*). In essence, within the framework of Eq. (1), local frequency shift mechanisms—such as those from chemical or microstructural origin—do not exist. Therefore, if a QSM algorithm based on Eq. (1) is applied to a frequency map with local frequency shifts, it misattributes these shifts to susceptibility variations in surrounding voxels. This misattribution results in non‐local artifacts in ***χ***, as illustrated in Figure 1 (right). These artifacts arise because the dipole convolution acts as a filter, modifying the power spectrum of **χ**. It attenuates spatial‐frequency components near the magic angle (approximately 54.7º relative to the B_0_ field direction).^66^ In contrast, the power spectrum of local contrast mechanisms is more like that of natural images, because it does not emerge from a convolution with a dipolar kernel. Natural images display isotropic, smoothly decaying energy with increasing frequency, typically following a power‐law behavior. In QSM, dipole inversion via deconvolution—solving Eq. (1)—recovers the attenuated frequency components by amplifying them. Unfittingly applied to local frequency contrast, this amplification overemphasizes spatial frequencies near the magic angle, resulting in a directional power spectrum with artificially high energy near that angle. This manifests as streaking artifacts in the reconstructed susceptibility maps.^6^, ^7^

## A.2. Power spectral densities

To confirm the similarities between the training data, the digital brain model, and the in vivo data, we calculated and visualized their power spectral densities (PSD = | FT(***f***) |^2^) (Figure A.1A). This also demonstrated the distinct characteristics between ***f***_***χ***_ and ***f***_***ρ***_.

Because the convolution ***d*** * ***χ*** in the spatial domain is equivalent to the point‐wise multiplication FT(***d***) · FT(***χ***) in the Fourier domain and ***d*** is Hermitian‐symmetric (i.e. FT(***d***) is real‐valued), the cone‐shaped pattern of PSD(***d***) is imprinted onto PSD(***f***). Accordingly, the PSDs from our simulated ***f***_***χ***_ (Figure A.1Ad,e) exhibit that cone pattern. In contrast, the PSDs from our simulated ***f***_***ρ***_ are isotropic. The PSDs of the simulated ***f*** (h, i) resemble those of the in vivo ***f*** (j), with a visible cone pattern.

## A.3. Training data synthesis

Our training data consisted of pseudo‐random patterns of **χ**, from which we calculated ***f***_***χ***_ via fast forward field computation.^9^ We generated a second set of pseudo‐random patterns, ***f***_***ρ***_, and calculated ***f*** as the sum of ***f***_***χ***_ and ***f***_***ρ***_. Figure 2C illustrates a typical synthetic sample. As a notable difference to Bollmann et al.,^29^ who used basic geometric shapes, such as cubes and spheres, our random pattern generator created more complex, organic‐looking patterns of various shapes and sizes with curved borders, nonuniform intensity, and complex texture. Our preliminary research indicated that careful design of the synthetic pseudo‐random distributions is essential to avoid biases and artifacts.^67^ Figure A.1B illustrates features of the pseudo‐random patterns. Without requiring explicit constraints to the power spectrum, this procedure yielded power spectral densities similar to in vivo data (Figure A.1). All details of the random pattern generator can be found in the published source code. To increase the training set size at minimal additional computational cost, the training data were randomly rotated (90° steps around the *z*‐axis of the static magnetic field), flipped (*x*, *y*, *z*), and scaled (factors 0.1 to 2 to facilitate linearity^68^), resulting in unique data at every iteration of the training. To increase the robustness against measurement noise,^69^ ***f*** was augmented with spatially variant, non‐Gaussian MR frequency noise (Figure 2C).^70^ First, we generated independent pseudo‐random magnitude distributions with values restricted between 0 and 1. During each training iteration, randomly drawn samples of these magnitude distributions were used to calculate complex‐valued MR signals, corresponding to ***f***. Specifically, real and imaginary parts of the signal were augmented with Gaussian noise with random standard deviations, and afterward, the phase was retrieved and converted back into frequency values resulting in unique noise patterns at every iteration. Finally, we applied random masks, consisting of a frayed hull at the edge of the volume and small spots within the volume, at locations with very low magnitude and, consequently, large phase noise.

## A.4. Neural network training

The network was trained with ADAM optimization^71^ (mean squared error loss function; learning rate of 3 × 10^−5^; batch size of 4; 10 000 epochs; 1200 base samples before augmentation; input and output size of 160^3^ voxels). The loss calculation included only the values within the masked region. To facilitate learning from samples with small values, and to reduce the effect of those with large values, each sample was assigned a weight based on its scaling factor during augmentation (1/scaling^2^). The state of the network's weights with the lowest loss on 120 held‐out samples was used for all applications. The training process took approximately 5 days on eight GPUs (NVIDIA RTX 2080 Ti). Inference with the trained network took less than 1 min per case on a state‐of‐the‐art CPU.

## A.5. Brain model creation

We created the microstructure effects for the model with microstructure according to Wharton and Bowtell,^7^ as proposed by the authors of the original challenge data set^40^:

(A.1)
$$f_{\rho }=f_{0}\left(-5\cdot \left(\sin^{2}\theta -\frac{2}{3}\right)\cdot \mathrm{FA}/0.59-3\right),$$

where *f*_0_ is the Larmor frequency of free water protons in the main magnetic field; FA is the fractional anisotropy from diffusion tensor imaging; and *θ* is the angle between the main magnetic field and the principal eigenvector of the diffusion tensor, reflecting the fiber orientation.
